# The Emerging Role of MicroRNAs in NAFLD: Highlight of MicroRNA-29a in Modulating Oxidative Stress, Inflammation, and Beyond

**DOI:** 10.3390/cells9041041

**Published:** 2020-04-22

**Authors:** Hung-Yu Lin, Ya-Ling Yang, Pei-Wen Wang, Feng-Sheng Wang, Ying-Hsien Huang

**Affiliations:** 1 Department of Internal Medicine, Kaohsiung Chang Gung Memorial Hospital and Chang Gung University College of Medicine, Kaohsiung 833, Taiwan; linhungyu700218@gmail.com (H.-Y.L.); wangpw@adm.cgmh.org.tw (P.-W.W.); 2 Center for Mitochondrial Research and Medicine, Kaohsiung Chang Gung Memorial Hospital and Chang Gung University College of Medicine, Kaohsiung 833, Taiwan; wangfs@ms33.hinet.net; 3 Department of Anesthesiology, Kaohsiung Chang Gung Memorial Hospital and Chang Gung University College of Medicine, Kaohsiung 833, Taiwan; inr453@cgmh.org.tw; 4 Genomics and Proteomics Core Laboratory, Department of Medical Research, Kaohsiung Chang Gung Memorial Hospital and Chang Gung University College of Medicine, Kaohsiung 833, Taiwan; 5 Department of Pediatrics, Kaohsiung Chang Gung Memorial Hospital and Chang Gung University College of Medicine, Kaohsiung 833, Taiwan

**Keywords:** microRNAs, microRNA-29a, NAFLD, NASH, liver fibrosis, mitochondria, inflammation

## Abstract

Non-alcoholic fatty liver disease (NAFLD) is a common cause of chronic liver disease and ranges from steatosis to steatohepatitis and to liver fibrosis. Lipotoxicity in hepatocytes, elevated oxidative stress and the activation of proinflammatory mediators of Kupffer cells, and fibrogenic pathways of activated hepatic stellate cells can contribute to the development of NAFLD. MicroRNAs (miRs) play a crucial role in the dysregulated metabolism and inflammatory signaling connected with NAFLD and its progression towards more severe stages. Of note, the protective effect of non-coding miR-29a on liver damage and its versatile action on epigenetic activity, mitochondrial homeostasis and immunomodulation may improve our perception of the pathogenesis of NAFLD. Herein, we review the biological functions of critical miRs in NAFLD, as well as highlight the emerging role of miR-29a in therapeutic application and the recent advances in molecular mechanisms underlying its liver protective effect.

## 1. Introduction

Non-alcoholic fatty liver disease (NAFLD), the most common cause of chronic liver disease, consists of fat deposited (steatosis) in the liver due to causes besides excessive alcohol use [1]. NAFLD is a hepatic manifestation of the metabolic syndrome [2] and is closely associated with cardio- and neurological complications, including cardiovascular disease, hypertension, cognitive dysfunction, and ischemic stroke [3,4]. NAFLD may result from “multiple and parallel hits” like lipotoxicity caused by the excessively elevated uptake of fatty acid (FA) into hepatocytes, subsequently elevated oxidative stress, and the activation of proinflammatory mediators of Kupffer cells (KC), as well as fibrogenic pathways of activated hepatic stellar cells (HSC), leading to non-alcoholic steatohepatitis (NASH) and non-alcoholic steatofibrosis (NASF) [5]. Considered a key predisposing factor for cirrhosis, NASH is implicated as a risk factor for non-viral hepatitis-related hepatocellular carcinoma (HCC) [6]. 

MicroRNAs (miRs) are small non-coding RNAs with about 22 nucleotides that have been found to have a regulatory role in transcriptional control mechanisms in guiding metabolic homeostasis. MiRs are first transcribed by RNA polymerase II or III in the nucleus to generate primary miRs (pri-miRs) which will be consequently processed to become precursor miRNAs (pre-miRs) [7]. The miRs then bind to 3′untranslated region (3′UTR) of target mRNA(s) and can lead to mRNA degradation or translational repression [8]. Despite of this dogmatic view in the repressive activity, some miRs acting to enhance gene expression has been recognized [9]. In addition, other mechanisms have recently been reported, such as miRNAs binding to 5′UTR or coding sequence of mRNA [10], Toll-like receptors [11,12] or mitochondrial transcripts [13]. There are increasing evidences reporting that some miRs regulate pathways governing lipid metabolism, oxidative stress and inflammation in the liver, playing a crucial role in the pathophysiology of NAFLD [14,15]. For example, miR-122, miR-34a, miR-33, miR-21, and miR-29 were acknowledged for their regulatory role in hepatic functions and for their therapeutic potential [14,15,16]. 

The miR-29 family in human includes miR-29a, -29b, and -29c. Mature miR-29s are highly similar between human, mouse, and rat [17]. Pathophysiological disturbance of various tissue or cell types could contribute to the alteration of circulating miR-29a level, as miR-29a level has been shown to corelate with diverse human diseases, including Alzheimer’s disease [18], Parkinson’s disease [19], ankylosing spondylitis [20], atherosclerosis [21], atrial fibrillation [22], active pulmonary tuberculosis [23], thoracic aneurysms [24], tendon disease [25], diabetes [26], scleroderma [27] and cholestatic pediatric liver disease [28]. 

In addition, miR-29a was tightly connected with diagnostic relevance of NAFLD [29], NASH [30], and liver fibrosis [30,31,32], as well as aggressiveness and prognosis of HCC [33,34,35,36]. Importantly, several lines of study have indicated the emerging roles of miR-29a through multiple signaling networks in experimental models simulating liver diseases, including NAFLD, NASH, fibrosis, and HCC (Table 1). In this review, we focus on the latest findings regarding the biological role of miR-29a, as well as some important miRs in NAFLD. We also discuss recent advances in the molecular mechanisms underlying miR-29a-mediated protective effects against hepatic dysfunction.

## 2. MiRs as Markers in Liver Disease

The liver serves as a central organ in energy metabolism as it mainly contributes to regulate absorptive glucose storage and post-absorptive glucose release, amino acid metabolism, and lipid/lipoprotein metabolism. Since Lee et al. firstly identified the critical function of miRs in regulating the development of C. *elegans* [57], mounting lines of evidence have revealed that miRs are pivotal for controlling metabolic homeostasis and represents relevance in diagnosing liver diseases. miR-122, which is the most abundant miRs in the liver, is involved in hepatic cholesterol and lipid metabolism [58], and presented an increased level in circulation in the context of NAFLD [59,60,61,62], making a well predictive panel when combined with miR-29a [29]. Moreover, dysregulated level of circulating miRs were also reported, including miR-122 [29], miR-34a [61,63], miR-33 [62], miR-21 [64], miR-192 [60], miR-221/222 [65], miR-375 [59], and miR-802 [66]. Table 2 exhibits a non-exhaustive list of miRs of important in the context of liver diseases.

In 2011, Roderburg et al. identified miR-29a as a non-invasive biomarker for liver fibrosis in cirrhosis patients and mouse model [32]. In the following year, Zhu et al. discovered the role of miR-29a as a prognostic marker in HCC patients receiving surgical resection [35]. At present, growing body of evidence has shown the significance of miR-29a as a diagnostic/prognostic tool in HCC [41,42,47,50,55,67,68,69,70] and alcoholic liver disease [71], as well as NAFLD. In 2017, Lopez-Riera et al. identified circulating miR-29a as one predictor of miRNAs biomarker set in NAFLD patients [72]. Studies from Lambrecht et al. as we as Jampoka et al. highlighted miR-29a as an essential part in predictive algorithm for NAFLD [29,31]. Furthermore, studies using mouse model also confirmed the close involvement of miR-29a in representing the progression of NAFLD [44,73] and NASH [74,75]. A non-exhaustive list of miR-29a with altered level in liver diseases is provided in Table 3.

**Table 2:** MiRs Implicated in Liver Diseases.

microRNA	Expression	Reference
miR-21	Up	[64]
miR-33	Up	[62]
miR-34a	Up	[61,63,64,76]
miR-103/107	Up	[77]
miR-122	Up	[29,59,60,61,63,76]
miR-132	Down	[78]
miR-146b	Down	[78,79]
miR-148a	Down	[80,81]
miR-181a	Up	[82,83,84]
miR-181d	Down	[79]
miR-192	Up	[59,60,63]
miR-197	Down	[79]
miR-221/222	Up	[65,85]
miR-375	Up	[59]
miR-802	Up	[66]

## 3. MiR-29a Functions as an Epigenetic Modifier to Mitigate Liver Injury

Epigenetic regulation acts to alter hereditary gene expression by modifying chromatin structure and the DNA methylation and acetylation that is not related to change the primary DNA sequence [86]. Unlike the genome of an organism, the epigenome is not a consistent entity and may be modulated by different intrinsic, chemical, and environmental cues [87], and its changes may be inherited across generations [88]. DNA methyltransferases (DNMTs), such as DNMT1, DNMT3a, and DNMT3b, enable DNA methylation, which is correlated with the conversion of quiescent HSC into hepatic myofibroblasts [89]. DNA methylation inhibitors exert a regulatory effect on hepatic wound healing and fibrogenesis [90,91]. Of particular note, our recent work demonstrated that miR-29a can repress DNMT3b expression by directly targeting its 3′UTR in the murine primary HSC [37]. Overexpression of miR-29a can alleviate steatosis, NASH, and NASF in methionine-choline-deficient diet-fed mice [37]. Another of our studies also showed that overexpression of miR-29a counteracts fibrosis in murine liver by reducing DNMT1 and DNMT3b [39], as well as the down-regulation of methyl CpG binding protein 2 (MeCP2) [38]. 

On the other hand, histone deacetylase (HDAC) 4, which acts to modify acetylation reactions in histones and non-histone proteins, has also been reported to have a positive role in activating liver fibrosis [92]. HDAC inhibitors administration exerts ameliorative effect both in experimental animal models and in in vitro cellular models of liver and kidney fibrosis [93]. In mechanistical term, miR-29a could exert suppressive effect on HDAC4 expression level by direct targeting 3′UTR of HDAC mRNA. Based on this molecular basis, our study demonstrated that overexpression of miR-29a in mice presents decreased HDAC4 activity and ameliorated liver fibrosis [40]. 

## 4. Role of miR-29a in Oxidative Stress and Inflammation

Under normal mitochondrial homeostasis conditions, physiological reactive oxygen species (ROS) can be effectively removed by antioxidant mechanisms and metabolic adaptations that inhibit substrate delivery to the tricarboxylic acid (TCA) cycle, a series of enzyme-catalyzed chemical reactions used by aerobic organisms to release energy [94]. However, in the context of NAFLD, both increased mitochondrial ROS production and the decreased activity of ROS scavenging mechanisms (e.g., glutathione, superoxide dismutase 2, and catalase) can increase oxidative stress, leading to lipid peroxidation, protein oxidation, and DNA oxidation, as well as mitochondrial damage [95,96]. Although the interaction between miR-29a and redox control in mitochondria is not fully understood, mice harbor miR-29a overexpression show mitigation of DNA oxidative damage and decreased stress-inducible marker heme oxidase-1 in NASH model, suggesting its role in neutralizing oxidative stress [37,97].

Elevated oxidative stress has been linked to altered mitochondrial membrane potential, as well as a loss of mitochondrial integrity in NAFLD. For example, cardiolipin, a unique phospholipid found in the inner mitochondrial membrane, is highly sensitive to oxidative stress, resulting in the induction of a mitochondrial permeability transition (MPT) pore opening, which has been suggested to provide routes for the cytosolic release of mitochondrial danger-associated molecular patterns (mtDAMPs) to trigger pro-inflammatory signaling [98,99,100]. For one, mtDNA that has escaped from mitochondria can activate innate immune signaling through NOD-like receptor family pyrin domain containing 3 (NLRP3) and toll-like receptors (TLRs) [101,102]. In high-fat diet (HFD)-fed mice, mtDNA has been shown to interact with the TLR9 on KC and HSC once released from damaged hepatocytes to trigger the signaling of innate immune and fibrogenesis, as has been suggested to occur in the pathogenesis of NASH [102,103,104]. The extracellular release of a mtDNA-associated protein, mitochondrial transcription factor A (TFAM), can also act as a mtDAMP to provoke pro-inflammatory macrophage activity [105]. mtdsRNA has been recently recognized as a novel mtDAMP that interacts with the dsRNA sensor to trigger innate immune signaling. In an alcoholic liver disease model, hepatocytes generate exosomal mtdsRNA to mediate TLR3 activation and subsequent IL-1β expression in KC [106]. Mitochondrial N-formyl peptides were released from hepatocyte trigger formyl peptide receptor 1 on KC, subsequently stimulating NF-κB activity [5]. Our recent work demonstrated that WT mice fed with HFD developed hepatic inflammation and fibrosis and had increased mtDNA and TFAM in the liver tissue, while those same phenomena in miR-29a transgene mice fed with HFD are greatly reduced [44], indicating that miR-29a may exert an anti-inflammatory effect on the pathogenesis of NAFLD by restricting mtDAMPs. Nevertheless, how miR-29a performs on the mitochondrial restrictive mechanism over these danger signal molecules warrants further study.

Some other miRs also present impacts on pathways mastering mitochondrial functions in the liver. MiR-122 was shown to be required for mitochondrial translation of respiratory proteins, improvement of mitochondrial respiratory enzyme activity and enhancement of mitochondrial proteostasis in the liver [107]. Inhibition of miR-34a was shown to mitigate steatosis in an experimental NAFLD model [108]. As miR-34a presents repressive activity on PPARα by direct association with its mRNA 3′UTR [108], the consequence of miR-34a inhibition could be mediated by an increase of PPARα level, resulting in increased mitochondrial biogenesis, decreased oxidative stress, and reduced inflammatory response. In a similar manner, miR-21 also play a negative role through suppresses PPARα. Inhibition of miR-21 restores PPARα level to exert protective effect on liver inflammation and fibrosis in an experimental mouse NASH [109].

## 5. Role of miR-29a in Mitochondrial Metabolism

Mitochondrial dysfunction in hepatocytes is a major hallmark of NAFLD [110,111]. While the exact mechanism underlying hepatic mitochondrial dysfunction during NAFLD is not fully understood, one possible reason may be that the HFD-caused flux of free FA into the liver leads to lowered mitochondrial respiratory chain activity, incomplete β-oxidation, decreased ATP synthesis, and dysregulated TCA cycle metabolism, all of which may bring about oxidative stress and lipotoxicity which can contribute to hepatic inflammation, insulin resistance and fibrogenic signaling [112,113].

CD36, an FA translocase, acts as a multifunctional membrane protein that facilitates the uptake of long-chain FA [114]. CD36 is a shared target of LXR, PXR, and PPAR-γ in their mediation of lipid homeostasis [115]. CD36 upregulation has been shown to be coupled with the blunted breakdown of hepatic triglyceride in mice fed an HFD, while CD36 also binds to ox-LDL in the liver [116]. Furthermore, higher plasma concentrations of CD36 were associated with body adiposity, reflecting more severe liver damage in NAFLD in humans [117]. The knockdown of CD36 contributes to improving lipid accumulation in the human hepatic cell line HepG2 [118], suggesting that the liver-specific knockout of CD36 decreases hepatic lipid levels, increases FA oxidation (FAO), and reduces liver inflammatory markers in HF diet-induced steatosis [119]. FAO primarily occurs in mitochondria, as well as in peroxisomes and cytochromes [120,121]. Mitochondrial dysfunction is an important feature of excessive FA influx, while increased FAO produces ROS and induces oxidative stress [122]. This imbalanced redox status can further promote damage to the mitochondrial membranes, leading to compromised liver function [123]. Cholesterol, especially oxidized (ox)-LDL, can recruit infiltrating macrophages and regenerate KC to contribute to the progression of NASH [124]. CD36-mediated ox-LDL also triggers CEBP-β expression to directly upregulate Nogo-B and promote lipophagy, causing lysophosphatidic acid-enhanced yes-associated protein 1 (YAP) oncogenic activity, which subsequently induces carcinogenetic signaling for NAFLD-associated HCC [2]. Ho et al. also revealed the periportal presents free cholesterol and ox-LDL accumulation that is associated with regional HSC activation and liver fibrosis in both human NAFLD [125] and mice [126]. Furthermore, Kawanishi et al. demonstrated that exercise training inhibits CD36 expression in KC and the liver of HFD and high-fructose water model mice [127]. 

In 2019, Chen et al. reported that ox-LDL/CD36 signaling in macrophage links dysregulated FA metabolism and oxidative stress from the mitochondria, which drove chronic inflammation in the atherosclerosis model [122]. Our group also discovered that miR-29a protects against glucocorticoid-mediated osteoporosis by suppressing the activity of osteoclasts and differentiating from macrophages [128], thus supporting its role in regulating immune cell activity. As a result, CD36 may play a role in linking FA metabolism with mitochondrial oxidative stress and inflammation, as well as be a promising target for reversing hepatic dysfunction in NAFLD. 

**Figure 1:**
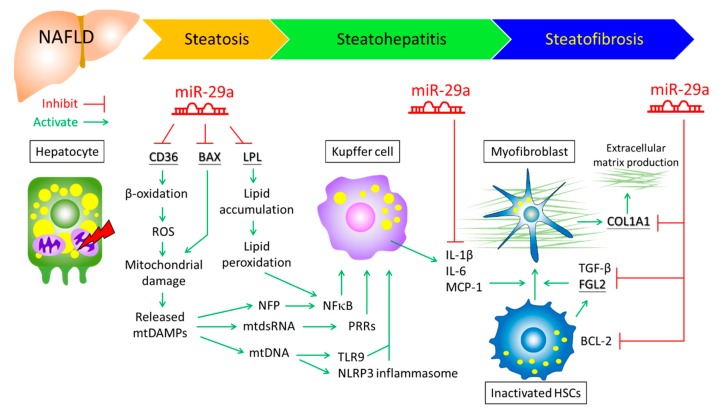
Role of miR-29a on modulating hepatic mtDAMPs release, lipid metabolism, inflammation, and fibrogenic signaling in the pathogenesis of NAFLD. Underlined genes represent miR-29a targets. BAX, Bcl-2-associated X protein; FGL2, fibrinogen-like 2; LPL, lipoprotein lipase; mtDAMPs, mitochondrial danger-associated molecular patterns; mtDNA, mitochondrial DNA; mtdsRNA, mitochondrial double-stranded RNA; NFP, N-formyl peptides; PRRs, pattern recognition receptors; NLRP3, NOD-like receptor family pyrin domain containing 3; TLR9, toll-like receptor 9.

On the bioinformatic basis that CD36 is a potential miR-29a target, our recent study further demonstrated that miR-29a in vitro directly binds to mRNA 3′UTR of CD36 and suppresses its expression in HepG2 cells [44]. Under HFD, wild type mice develop steatosis and steatofibrosis and show an increased hepatic CD36 level, as well as elevated mtDAMPs and pro-inflammatory genes, while these phenotypes and markers are significantly reduced in miR-29aTg mice [44]. These findings support that lipid accumulation and hepatic inflammation could be effective counteracted on the basis of modulating the miR-29a/CD36/mitochondria axis (Figure 1). Nevertheless, the role of miR-29a in individual cell types (hepatocyte, KC, and HSC) and how it regulates intercellular cross-talk requires further study.

In addition to nutrient metabolism, the dynamic properties of mitochondria—including their fusion, fission, and degradation—are vital for their optimal function and quality control [129]. Recent studies have suggested that mitochondrial dynamics and quality control mechanism mitophagy play a key role in NAFLD [130]. Several lines of evidence have revealed that miR-29a can modulate mitochondrial homeostasis by directly targeting key genes. With regard to protecting mitochondrial structural integrity, miR-29a can target voltage-dependent anion channel [131] and Bcl-2-associated X (BAX) genes [132], whose oligomerization is involved in MPT pore opening and mtDAMPs release [100,133]. The p53 upregulated modulator of apoptosis (PUMA), an activator of BAX/BAK for the induction of MPT and subsequent apoptosis, can also be targeted by miR-29a [134]. Moreover, one computational analysis revealed that miR-29a targets dynamin-related protein 1 (DRP1) [135], implying its potential role in regulating mitochondrial dynamics and mitophagy. In line with the aforementioned studies, our experimental results have shown that liver protective-miR-29a can reduce BAX expression [46]. More in-depth studies are warranted to investigate the therapeutic potential of miR-29a and shed light on the sophisticated interplay between miR-29a, mitochondrial repurposing, and inflammatory signaling.

In this regard, miR-34a/SIRT1/AMPK pathway was shown to cause mitochondrial dynamics dysfunction in mouse NASH model [136]. Another late study presented that miR-34a impair mitochondria quality control mechanism through Sirt3/FoxO3a/PINK1 signaling in an experimental mouse model of liver inflammation [137].

## 6. MiRNAs Involved in Lipid Metabolism of NALFD

In the context of NAFLD, dysregulated miRs critically contribute to perturb pathways for lipid metabolism, including (i)synthesis of FA, triglycerides, and cholesterol, (ii) uptake of lipid in the blood, (iii) hepatic export of lipid and (iv) lipid oxidation [14]. These aberrances could aggravate steatosis and impair cellular redox, leading to lipid peroxidation [138]. Furthermore, lipid peroxidation-produced l 4-hydroxy-trans-2-nonenal (HNE) was shown to activate proinflammatory transcription factor NF-κB [139]. In this regard, miR-122 which is the most abundant miRs in the liver was recognized as a prominent example in these metabolic processes. Overexpression of miR-122 was shown to counteract lipid accumulation in cultured hepatocytes or in the liver of NASH mouse model through several mechanisms, such as: (i) Yin Yang 1, farnesoid X receptor, and small heterodimer partner (YY1/FXR/SHP) axis [140]; (ii) hypoxia-inducible factor-1alpha, vimentin, and mitogen-activated protein kinase kinase kinase 3 (HIF-1α/vimentin/MAP3K3) axis [141]; (iii) hepatocyte nuclear factor 4α (HNF4α) pathway [142]; stearoyl-CoA desaturase gene (SCD) [143].

In addition, the key role of miR-21 in hepatic lipid metabolism has recently been highlighted [14,144]. miR-21 can target phosphatase and tensin homolog (PTEN), which prevents hepatic steatosis, and PPARα, which activates lipid oxidation. Hepatocyte-specific knockout of miR-21 in mice improved HFD-induced steatosis through upregulation of multiple miR-21-targeted pathways governing lipid metabolism [145]. In lung cancer cells, inhibition of miR-21 was shown to restrain FA uptake by down-regulating CD36 protein expression [146]. 

MiR-33 is intimately implicated in both cholesterol and FA metabolism. In hepatic cell lines, miR-33 targets ABCA1 and ABCG1, which are cholesterol efflux regulatory proteins, and carnitine Palmitoyltransferase 1A (CPT1A) and AMPKα [147,148], which regulate FA β-oxidation. However, the effect of miR-33 in the pathogenesis of NAFLD appears to be debatable. Horie et al. showed that deletion of miR-33 in mice results in aggravating obesity and liver fibrosis induced by HFD via targeting sterol regulatory element-binding protein 1 (SREBP1) [149]. As well, Price et al. demonstrated that abrogation of miR-33 promotes obesity and insulin resistance [150]. On the contrary, Karunakaran et al. presented therapeutic effect of miR-33 inhibitor on promoting FA oxidation and preventing atherosclerosis in mice [151]. 

Of particular note, the central role of miR-29a in lipid metabolism was identified by Mattis et al. Their study conducted a bias-free, hepatocyte-specific global miRNAs deficiency in mouse and combined gene/miR profiling, demonstrating that miR-29a acts to prevent lipid accumulation in the liver by targeting lipoprotein lipase (LPL) 3′UPR [152]. These evidences underscore miR-29a/LPL axis’s close involvement in the reprogramming of lipid distribution in the liver, which may account for preventing steatosis-steatohepatitis transition (Figure 1). 

## 7. The Role of miR-29a in Fibrogenesis

Stressed hepatocyte release mtDAMPs, which leads to KC activation. Inflammatory mediators secreted by activated KC trigger activation of HSC. HSC activation consequently plays a central role for liver fibrogenesis, because these cells transdifferentiate into myofibroblasts and represent the major extracellular matrix producing cells [5,153,154]. Free cholesterol accumulation caused-lipotoxicity sensitizes HSCs to TGFβ-induced activation through TLR4 signaling in NASH mouse model [155]. Our previous work showed that overexpression of miR-29a exerts anti-fibrotic effect in BDL mouse model, and acts to reduce fibrogenesis by down-regulating COL1A1 and induce HSC apoptosis by enhancing PTEN [39]. Meanwhile, our findings revealed that miR-29a also regulates innate immune response through surpassing pattern recognition receptors TLR2/TLR4 in HSC. This effect in vivo contributes to a reduction of MyD88 and NK-kB, leading to decreased proinflammatory cytokines [45]. In NASH mouse model, we demonstrated that miR-29aTg provides protective effect through suppressing TGF-β and SMAD3 [37], two critical positive regulator of HSC activation [156]. Furthermore, we recently uncovered that anti-fibrotic effect of miR-29a is associated with inhibition of bromodomain-containing protein 4 (BRD4) in HSC [38], which represents a novel therapeutic target of liver fibrosis [157]. In view of using miR-29a as an interventional approach, Matsumoto et al. have demonstrated that administration of miR-29a can reverse liver fibrosis in CCl_4_− and thioacetamide-treated mice. Mechanical study revealed that miR-29 in HSC inhibits extracellular matrix production via targeting COL1A1 and FGL2, proliferation via targeting mitogen-activated protein kinase kinase kinase kinase 4 (MAP4K4) and platelet-derived growth factor C (PDGFC). It also acts to anti-inflammation and pro-apoptosis through repressing IL-1β and BCL-2 [48]. In addition, Knabel et al. demonstrated that administration of adeno-associated virus (AAV) serotype 8-encoded miR-29a prevents CCl_4_-induced liver fibrosis [158]. 

In contrast to miR-29a, studies with reference to miR-34a is still in dispute. MiR-29a was reported to exert detrimental effect in promoting HSC activation and liver fibrosis via targeting Sirt1 [159], while another study demonstrated that overexpression of miR-34 in HSCs ameliorated the development and progression of liver fibrosis by targeting Smad4 and regulating TGF-β1/Smad3 pathway [160]. In addition, exogenous mir-122 was shown to exerts inhibitory effect on mouse liver fibrosis and HSC activation via a mutual modulation with sterol regulatory element-binding protein-1c (SREBP-1c) [161].

## 8. Conclusions

NAFLD can be caused by lipid dysregulation in hepatocytes, elevated ROS, and the activation of proinflammatory mediators of KC, as well as fibrogenic pathways of activated HSC, thus leading to NASH and NASF and rendering a predisposed milieu for cirrhosis and HCC. Therefore, curative approaches focused on modulating epigenetic modification and the inhibition of metabolic damage, oxidative stress, inflammation, and fibrogenic signaling to treat NAFLD is vital but challenging. As such, the role of miR-29a is emerging because its versatile function on epigenetic activity, mitochondrial homeostasis, and immunomodulation may improve our understanding of NAFLD (Figure 1). Increasing evidence has emphasized that miR-based therapeutic tools have potential and the significance of miR-29a is emerging, paving an innovative path for the future treatment of NAFLD.

## Figures and Tables

**Table 1:** Pleiotropic Role of miR-29a in in Liver Disease.

Affected Pathway	Disease Model	miR-29a Targets	References
**Epigenetics**	NASH, liver fibrosis, HCC	DNMT3b, HDAC4, DNMT3a, TET1	[37,38,39,40,41,42,43]
**Oxidative stress/Inflammatory**	NASH, liver fibrosis, HCC	CD36, DNMT3b, HDAC4, ARRB1, PTEN	[37,40,44,45,46,47,48,49]
**Apoptosis**	liver fibrosis, HCC	COL1A1, FGL2, MAP4K4, PDGFC, BCL-2, DNMT3a, MCL-1	[42,46,48,50,51,52]
**Autophagy**	NASH, liver fibrosis, HCC	DNMT3b, SPARC	[36,37,51]
**Epithelial-mesenchymal transition**	NASH, liver fibrosis	COL1A1, FGL2, MAP4K4, PDGFC	[37,39,40,44,45,46,48,51,53]
**Cell cycle**	HCC	SIRT1; SPARC; HULC, TET1, TET2, TET3	[36,41,43,54,55]
**Cell migration**	HCC	CLDN1, TET1, TET2, TET3, PTEN	[41,43,56]

**Table 3:** Clinical Relevance of miR-29a in the Diagnosis of Liver Disease.

Source	Expression	Clinical Relevance	Reference
Plasma	Down	Biomarker implicated in miRFIB scoring algorithm for diagnosis of liver fibrosis	[31]
Serum	Down	Reduced miR-29a along with elevated miR-122 serve as a diagnostic panel for NAFLD	[29]
Serum	Down	negatively correlated with necroinflammation and liver fibrosis	[30]
Serum	Down	Biomarker of advanced liver cirrhosis	[32]
Serum	Up	Biomarker of HCC	[67]
Plasma	Down	Prognostic marker of poor outcome of HCC	[33]
Serum	Up	Predictor for poor survival of HCC	[70]
HCC tissue	Up	Predictor for recurrence of HCC	[35]
HCC tissue	Down	Prognostic marker of poor outcome of HCC	[55]
HCC tissue	Down	Predictor for low survival rate of HCC	[36]
